# Modern imaging in Cushing’s disease

**DOI:** 10.1007/s11102-022-01236-w

**Published:** 2022-06-06

**Authors:** W. A. Bashari, D. Gillett, J. MacFarlane, A. S. Powlson, A. G. Kolias, R. Mannion, D. J. Scoffings, I. A. Mendichovszky, J. Jones, H. K. Cheow, O. Koulouri, M. Gurnell

**Affiliations:** 1 Cambridge Endocrine Molecular Imaging Group, Metabolic Research Laboratories, Wellcome–MRC Institute of Metabolic Science, University of Cambridge and National Institute for Health Research Cambridge Biomedical Research Centre, Addenbrookeʼs Hospital, Cambridge Biomedical Campus, Cambridge, CB2 0QQ UK; 2 Department of Nuclear Medicine, Addenbrookeʼs Hospital, Cambridge Biomedical Campus, Cambridge, CB2 0QQ UK; 3 Department of Neurosciences, University of Cambridge, Cambridge Biomedical Campus, Cambridge, CB2 0QQ UK; 4 Department of Neurosurgery, Addenbrookeʼs Hospital, Cambridge Biomedical Campus, Cambridge, CB2 0QQ UK; 5 Department of Radiology, Addenbrookeʼs Hospital, Cambridge Biomedical Campus, Cambridge, CB2 0QQ UK

**Keywords:** Pituitary Cushing's, MRI, Molecular / functional imaging, PET

## Abstract

Management of Cushing’s disease is informed by dedicated imaging of the sella and parasellar regions. Although magnetic resonance imaging (MRI) remains the investigation of choice, a significant proportion (30–50%) of corticotroph tumours are so small as to render MRI indeterminate or negative when using standard clinical sequences. In this context, alternative MR protocols [e.g. 3D gradient (recalled) echo, with acquisition of volumetric data] may allow detection of tumors that have not been previously visualized. The use of hybrid molecular imaging (e.g. ^11^C-methionine positron emission tomography coregistered with volumetric MRI) has also been proposed as an additional modality for localizing microadenomas.

## Introduction

The sense of achievement that accompanies successful navigation of the first phase of investigation in Cushing’s syndrome (CS) is often tempered by the knowledge that localizing the source in ACTH-dependent disease may represent an even greater challenge due to the occult nature of many corticotroph and neuroendocrine tumors [1, 2]. However, help is at hand, with several recent advances in MRI, CT (computed tomography) and PET, facilitating the successful detection of tumors that may be only a few millimeters in diameter. Here, we outline a stepwise approach to modern imaging in suspected pituitary-dependent CS.

## Pituitary MRI

Most corticotroph tumors are microadenomas (even ‘picoadenomas’) and many (up to 50%) are not readily visualized using lower field strength [1.5 Tesla (1.5 T)] MRI, especially if acquired using 2–3 mm slice thickness with gaps between consecutive slices. A tiered approach to sellar and parasellar MRI is therefore recommended, with early onward referral to a pituitary tumor center of excellence (PTCoE), especially when initial MRI findings are inconclusive [1–3].

### Step 1a: core protocol [conventional spin echo (SE) MRI]

- Coronal and sagittal T1-weighted (T1w) SE pre- and post-gadolinium
- Coronal T2w fast (turbo) spin echo (FSE/TSE)

Both sequences should be acquired with 2 (maximum 3) mm slice thickness and minimal slice spacing, using 3 T MRI [1, 2, 4]. For corticotroph macroadenomas (~ 10–20%) of all corticotroph tumors, T2w sequences can provide useful information regarding the potential invasion of adjacent parasellar structures and may also reveal a micro- or macrocystic appearance [1, 2].

### Step 1b: recommended supplementary sequences

If the core protocol does not identify a macroadenoma or obvious microadenoma, consider proceeding immediately (ideally in the same session) to:

- T1w gadolinium enhanced 3D-spoiled gradient (recalled) echo (3D-SGE/3D-GRE) MRI:
  - this allows volumetric (1 mm slice thickness) data acquisition, to provide better soft tissue contrast and improved detection of smaller lesions
  - and has been reported to localize up to 80–90% of corticotroph tumors [5, 6].
- T1w gadolinium-enhanced dynamic MRI (dMRI):
  - which involves repeated data acquisitions every 10–20 s over 1–2 min, commencing with contrast injection (microadenomas show delayed enhancement during early phase):
  - however, although Liu and colleagues recently reported a high positive predictive value when dMRI was combined with high dose dexamethasone suppression testing [7], several groups have suggested that dMRI is inferior to SGE/GRE MRI and frequently yields false positive findings [1, 2, 6].

### Step 2: optional supplementary sequences/magnetic field strength

When doubt remains as to the location of a corticotroph microadenoma, additional MR sequences or a higher magnetic field strength may be considered [1, 2]:

- Fluid-attenuated inversion recovery (FLAIR) with gadolinium enhancement
  - to detect delayed contrast washout in a microadenoma [8].
- Constructive interference in steady state (CISS)
  - a high spatial resolution fast T2w gradient echo sequence, which allows fast acquisition times, high signal-to-noise ratio, and improved contrast-to-noise ratio [9].
- Isotropic 3D-fast (turbo) SE (e.g. *SPACE, Cube, VISTA, isoFSE, 3D MVOX*)
  - which produces high resolution 3D images (with features of T1w, T2w and proton density MRI) [10].
- Ultra-high field (7 T) MRI

Whichever sequences are deployed, access to an expert neuroradiologist, supported by a specialist pituitary multidisciplinary team, is critical to maximizing the chance of localizing the causative lesion, whilst avoiding false attributions to artifacts or incidental lesions [2].

## Pituitary PET

Even in centers with access to the full range of MR sequences, structural imaging may return indeterminate or negative results. The adoption of higher resolution techniques also increases the chance of detecting incidental lesions. In these contexts, molecular (functional) imaging can confirm or refute the site of a suspected microadenoma or reveal a previously unsuspected abnormality. Several radioligands have been used with varying degrees of success and are summarized here.

### ^11^C-methionine PET (Met-PET)

**Fig. 1:**
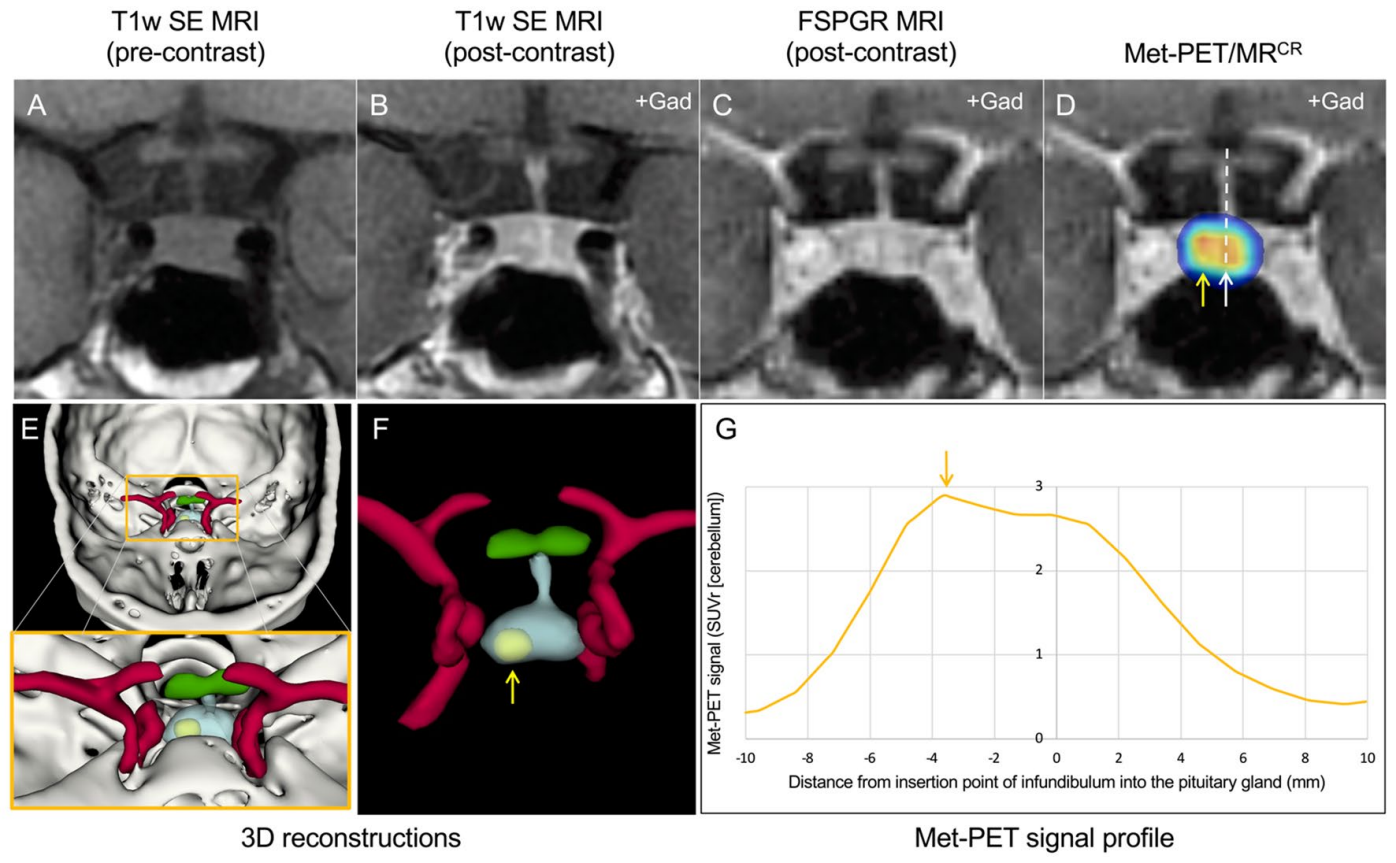
MRI and Met-PET findings with 3D reconstruction of the sella and parasellar regions in a patient with ACTH-dependent Cushing’s syndrome. **A–C** Pre- and post-contrast coronal T1w SE MRI (**A**, **B**) and FSPGR (volumetric) MRI (**C**) demonstrate equivocal appearances, with subtle deviation of the infundibulum to the left, but minor downward sloping of the floor of the sella on the left side. No discrete microadenoma is readily visualized. **D** Met-PET/MR^CR^ reveals both central (white arrow) and right-sided (yellow arrow) radiotracer uptake in the gland. **E**, **F** 3D reconstructed images, combining PET, CT and FSPGR MRI datasets, allows appreciation of the location of the tumor (yellow) with respect to the normal gland (turquoise) and other adjacent structures including the intracavernous carotid arteries (red) and optic chiasm (green). **G** Profiling of ^11^C-methionine uptake across the sella reveals two peaks consistent with uptake by normal gland and a corticotroph microadenoma. At transsphenoidal surgery, a microadenoma was resected from the right side of the gland and confirmed histologically to be a corticotroph adenoma. Postoperatively the patient achieved complete clinical and biochemical remission and remains eupituitary. Key: *CT *computed tomography; *FSPGR* fast spoiled gradient recalled echo; *Gad* gadolinium; *Met*-*PET*/*MR*^*CR*^ ^11^C-methionine PET-CT coregistered with volumetric (FSPGR) MRI; *MRI* magnetic resonance imaging; *PET* positron emission tomography; *SE* spin echo; *T*1*w* T1-weighted

The introduction of hybrid imaging techniques [Met-PET/MR or Met-PET/CT coregistered with volumetric MRI (Met-PET/MR^CR^)] has allowed several groups to demonstrate the utility of combining anatomical and functional imaging with ^11^C-methionine to localize small corticotroph adenomas [11–13]. This approach has been successfully used in both de novo and recurrent disease [13], and the development of enhanced image analysis tools, together with algorithms for 3D reconstruction and sellar profiling, offer the potential to further increase confidence in localizing very small tumors (Fig. 1).

### ^18^F-FET PET

A key limitation of Met-PET is the short half-life (~ 20 min) of ^11^C-methionine, which restricts its use to centers with an on-site cyclotron. In contrast, ^18^F-fluoroethyltyrosine (FET) with its longer half-life (~ 110 min) can be synthesized and then transported to off-site centers. Both methionine and fluoroethyltyrosine are taken up at sites of peptide synthesis, possibly via the same l-type amino acid transporter (LAT1). To date, only a small number of patients with Cushing’s disease have been imaged using ^18^F-FET-PET/MR, but initial findings suggest a high predictive value for localizing corticotroph microadenomas [12].

### ^18^F-FDG PET

Although ^18^F-fluorodeoxyglucose (^18^F-FDG) shares the benefits of a longer half-life and is more widely available, studies of ^18^F-FDG PET in Cushing’s disease have proved largely disappointing, with no clear benefits over conventional MRI in most published series. However, Boyle and colleagues have proposed that prior corticotropin releasing hormone (CRH) injection may increase the sensitivity of ^18^F-FDG PET in Cushing’s disease [14].

### ^68^Ga(-DOTA)-CRH PET

Recognizing that most corticotroph adenomas express CRH receptors (CRH-1R), Walia and colleagues observed that conjugation of ^68^Ga-DOTA to CRH (^68^Ga-CRH) yields a PET ligand with apparent high sensitivity and specificity for the detection of ACTH-secreting pituitary adenomas [15]. However, importantly, only 10 of 24 subjects had adenomas < 6 mm in size and in only four subjects was a lesion not visualized on MRI [15].

### ^68^Ga(-DOTA)-SSTR PET

Although corticotroph adenomas can express somatostatin receptor (SSTR) subtypes 2A, 3 and 5, the use of ^68^Ga-labelled SSTR ligands is largely reserved for the localization of neuroendocrine tumors (NETs) in the ectopic ACTH syndrome [1].

## Conclusions

As set out in the recent Pituitary Society guideline update [3], MRI remains the imaging modality of choice for the localization of ACTH-secreting pituitary adenomas and, when conducted in a specialist unit with access to the full complement of sequences, will identify the causative lesion in many cases. However, when uncertainty persists, molecular PET imaging may allow the causative lesion to be located.

## References

[1] Senanayake R, Gillett D, MacFarlane J, Van de Meulen M, Powlson A, Koulouri O (2021). New types of localization methods for adrenocorticotropic hormone-dependent Cushing’s syndrome. Best Pract Res Clin Endocrinol Metab.

[2] Bonneville J, Potorac I, Petrossians P, Tshibanda L, Beckers A (2022). Pituitary MRI in Cushing’s disease-an update. J Neuroendocrinol.

[3] Fleseriu M, Auchus R, Bancos I, Ben-Shlomo A, Bertherat J, Biermasz NR (2021). Consensus on diagnosis and management of Cushing’s disease: a guideline update. Lancet Diabetes Endocrinol.

[4] Erickson D, Erickson B, Watson R, Patton A, Atkinson J, Meyer F (2010). 3 Tesla magnetic resonance imaging with and without corticotropin releasing hormone stimulation for the detection of microadenomas in Cushing’s syndrome. Clin Endocrinol (Oxf).

[5] Kasaliwal R, Sankhe SS, Lila AR, Budyal SR, Jagtap VS, Sarathi V (2013). Volume interpolated 3D-spoiled gradient echo sequence is better than dynamic contrast spin echo sequence for MRI detection of corticotropin secreting pituitary microadenomas. Clin Endocrinol (Oxf).

[6] Grober Y, Grober H, Wintermark M, Jane JA, Oldfield EH (2018). Comparison of MRI techniques for detecting microadenomas in Cushing’s disease. J Neurosurg.

[7] Liu Z, Zhang X, Wang Z, You H, Li M, Feng F (2020). High positive predictive value of the combined pituitary dynamic enhanced MRI and high-dose dexamethasone suppression tests in the diagnosis of Cushing’s disease bypassing bilateral inferior petrosal sinus sampling. Sci Rep.

[8] Chatain GP, Patronas N, Smirniotopoulos JG, Piazza M, Benzo S, Ray-Chaudhury A (2018). Potential utility of FLAIR in MRI-negative Cushing’s disease. J Neurosurg.

[9] Lang M, Habboub G, Moon D, Bandyopadhyay A, Silva D, Kennedy L (2018). Comparison of constructive interference in steady-state and T1-weighted MRI sequence at detecting pituitary adenomas in Cushing’s disease patients. J Neurol Surg B.

[10] Wu Y, Cai Y, Rui W, Tang Y, Yang Z, He M (2022). Contrast-enhanced 3D–T2-weighted SPACE sequence for MRI detection and localization of adrenocorticotropin (ACTH)-secreting pituitary microadenomas. Clin Endocrinol (Oxf).

[11] Ikeda H, Abe T, Watanabe K (2010). Usefulness of composite methionine-positron emission tomography/3.0-tesla magnetic resonance imaging to detect the localization and extent of early-stage Cushing adenoma. J Neurosurg.

[12] Berkmann S, Roethlisberger M, Mueller B, Christ-Crain M, Mariani L, Nitzsche E (2021). Selective resection of cushing microadenoma guided by preoperative hybrid 18-fluoroethyl-L-tyrosine and 11-C-methionine PET/MRI. Pituitary.

[13] Koulouri O, Steuwe A, Gillett D, Hoole AC, Powlson AS, Donnelly NA (2015). A role for 11C-methionine PET imaging in ACTH-dependent Cushing’s syndrome. Eur J Endocrinol.

[14] Boyle J, Patronas NJ, Smirniotopoulos J, Herscovitch P, Dieckman W, Millo C (2019). CRH stimulation improves 18F-FDG-PET detection of pituitary adenomas in Cushing’s disease. Endocrine.

[15] Walia R, Gupta R, Bhansali A, Pivonello R, Kumar R, Singh H (2021). Molecular imaging targeting corticotropin-releasing hormone receptor for corticotropinoma: a changing paradigm. J Clin Endocrinol Metab.

